# Melodic Intonation Therapy in Chronic Aphasia: Evidence from a Pilot Randomized Controlled Trial

**DOI:** 10.3389/fnhum.2016.00533

**Published:** 2016-11-01

**Authors:** Ineke Van Der Meulen, Mieke W. M. E. Van De Sandt-Koenderman, Majanka H. Heijenbrok, Evy Visch-Brink, Gerard M. Ribbers

**Affiliations:** ^1^Rijndam Rehabilitation InstituteRotterdam, Netherlands; ^2^Department of Rehabilitation Medicine, Erasmus MC University Medical CentreRotterdam, Netherlands; ^3^Department of Neurology, Erasmus MC University Medical CentreRotterdam, Netherlands

**Keywords:** aphasia, stroke rehabilitation, language therapy, melodic intonation therapy, effectiveness

## Abstract

Melodic Intonation Therapy (MIT) is a language production therapy for severely non-fluent aphasic patients using melodic intoning and rhythm to restore language. Although many studies have reported its beneficial effects on language production, randomized controlled trials (RCT) examining the efficacy of MIT are rare. In an earlier publication, we presented the results of an RCT on MIT in subacute aphasia and found that MIT was effective on trained and untrained items. Further, we observed a clear trend in improved functional language use after MIT: subacute aphasic patients receiving MIT improved considerably on language tasks measuring connected speech and daily life verbal communication. Here, we present the results of a pilot RCT on MIT in *chronic* aphasia and compare these to the results observed in subacute aphasia. We used a multicenter waiting-list RCT design. Patients with chronic (>1 year) post-stroke aphasia were randomly allocated to the experimental group (6 weeks MIT) or to the control group (6 weeks no intervention followed by 6 weeks MIT). Assessments were done at baseline (T1), after 6 weeks (T2), and 6 weeks later (T3). Efficacy was evaluated at T2 using univariable linear regression analyses. Outcome measures were chosen to examine several levels of therapy success: improvement on trained items, generalization to untrained items, and generalization to verbal communication. Of 17 included patients, 10 were allocated to the experimental condition and 7 to the control condition. MIT significantly improved repetition of trained items (β = 13.32, *p* = 0.02). This effect did not remain stable at follow-up assessment. In contrast to earlier studies, we found only a limited and temporary effect of MIT, without generalization to untrained material or to functional communication. The results further suggest that the effect of MIT in chronic aphasia is more restricted than its effect in earlier stages post stroke. This is in line with studies showing larger effects of aphasia therapy in earlier compared to later stages post stroke. The study was designed as an RCT, but was underpowered. The results therefore have to be interpreted cautiously and future larger studies are needed.

Clinical Trial Registration: www.ClinicalTrials.gov, identifier NTR 1961.

## Introduction

Aphasia is a language disorder resulting from brain damage. It is a heterogeneous phenomenon, varying from mild word retrieval difficulties to a complete inability to produce and understand language. A wide variety of language treatments is available with a growing body of evidence (Robey, 1998; Cicerone et al., 2005; Rohling et al., 2009; Brady et al., 2012; Cotoi et al., 2016). Aphasia is often subdivided into fluent and non-fluent aphasia. Non-fluent aphasia generally results from a stroke in left fronto-temporal regions and is characterized by slow, effortful speech. Language production in these patients is mostly restricted to one- or two-word utterances. Despite their severe language production impairment, non-fluent aphasic patients are able to sing fluently, which has led to the use of singing and music in aphasia therapy.

One of the most formalized aphasia treatment methods using music is Melodic Intonation Therapy (MIT) (Albert et al., 1973). In MIT, ‘musical’ elements of language, such as rhythm and intonation, are used to facilitate and improve language production. Patients repeat short melodically intoned utterances (Albert et al., 1973; Helm-Estabrooks et al., 1989; Sparks, 2008). The therapy includes several therapeutic techniques, such as left hand-tapping and reducing speech rate. Gradually, the speech-language therapist (SLT) provides less support, until a patient is able to produce a trained utterance independently. MIT aims to improve connected speech.

The original American MIT has been translated into several languages, including French (Zumbansen et al., 2014), Persian (Bonakdarpour et al., 2003), Dutch (Van der Lugt-van Wiechen and Verschoor, 1987), and Caucasian (Breier et al., 2010). It is used by SLTs world-wide and many studies have reported its beneficial effects on language production (Sparks et al., 1974; Goldfarb and Bader, 1979; Carlomagno et al., 1997; Wilson et al., 2006; Schlaug et al., 2008, 2009; Hough, 2010). However, most of these studies were case studies or case series in chronic aphasia (Van der Meulen et al., 2012). To our knowledge, there are three non-randomized group studies on MIT in chronic aphasia. In two of them, data from a control group are lacking: Bonakdarpour et al. (2003) examined the effect of MIT in a group of seven Persian-speaking individuals with chronic non-fluent aphasia and observed improved performance on several language tasks as well as in connected speech. Cortese et al. (2015) reported improved language production in six chronic Italian aphasic patients after MIT as well as on follow-up assessments 6 months later. A third, recent group study was done by Wan et al. (2014). They examined the effects of MIT in a group of 11 chronic non-fluent English-speaking aphasic patients and reported improved communicative effectiveness and verbal fluency after MIT, associated with structural changes in the white matter underlying the right inferior frontal gyrus. In the untreated control group (*n* = 9), no changes in right hemisphere brain regions and no language improvement were observed. However, they do not report any statistical analyses comparing therapy success in the treated and untreated groups. In 1994, the American Academy of Neurology classified MIT as a promising therapy with a poor level of evidence (American Academy of Neurology, 1994). Despite its widespread use, the quality of the evidence remains poor (Hurkmans et al., 2012; Van der Meulen et al., 2012).

We performed a randomized controlled trial (RCT) to evaluate the efficacy of MIT in subacute aphasia. The results of the trial in subacute aphasia have been published earlier (Van der Meulen et al., 2014). In summary, in subacute severely non-fluent aphasic patients MIT yielded improved repetition of trained as well as untrained utterances. In addition, we found indications of a generalization toward improved verbal communication in daily life. Thirdly, treatment intensity and time post stroke were related to MIT success, but no patient-related determinants were observed.

In the present study, we used the same study design to evaluate MIT efficacy in chronic aphasia and examine whether the results observed in subacute aphasia could be replicated in a chronic aphasic population.

## Materials and Methods

### Design

**FIGURE 1:**
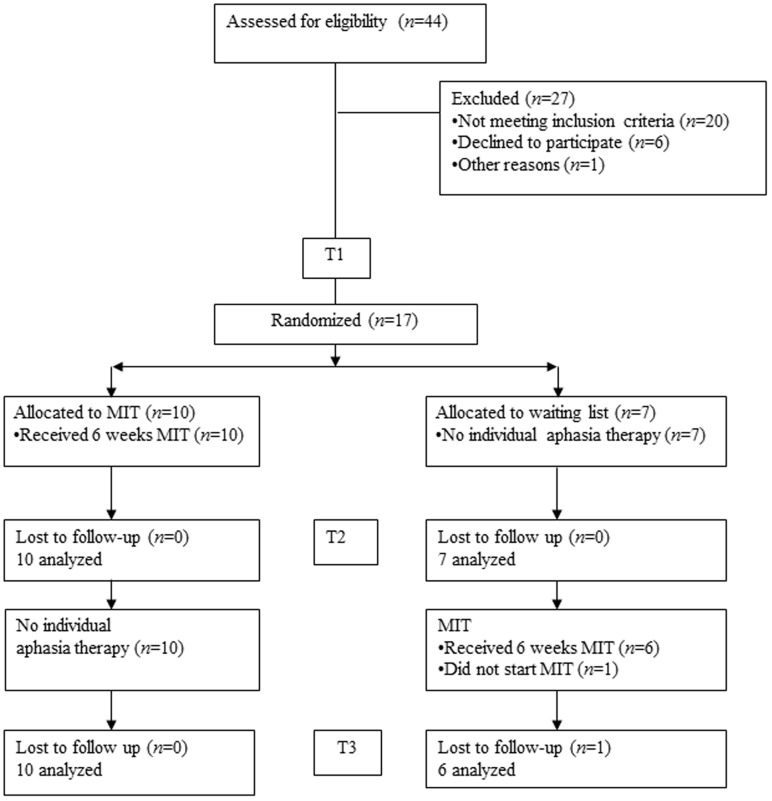
**Flow chart**.

The study was a multicenter waiting-list observer-blinded RCT with patients randomly allocated to either the experimental or the control group (**Figure 1**). We used a computer-generated allocation sequence, placed in consecutively numbered sealed opaque envelopes. Between T1 and T2, patients assigned to the experimental group received intensive MIT (6 weeks, 5 h/week). Patients in the control group received no individual aphasia treatment. To improve adherence to the program and prevent drop out, the control group received 6 weeks intensive MIT (5 h/week) between T2 and T3, i.e., after the waiting period of 6 weeks. The experimental group received no treatment between T2 and T3 (**Figure 1**). Efficacy of MIT was evaluated at T2, where language improvement after MIT was compared to improvement in the untreated control group.

The study was approved by the Medical Ethics Committee of Erasmus University Medical Center. All participants or a close relative gave written informed consent. For obvious reasons, patients and therapists could not be blinded to treatment conditions. The researchers assessing and scoring the tests were blinded for group allocation and test moment.

### Participants

Between 2009 and 2011, patients were recruited through the Dutch National Association of Persons with Aphasia and from several outpatient aphasia centers in the Netherlands. Inclusion criteria were: MIT candidate, right-handed before stroke, >1 year post stroke, age 18–80 years, and native language Dutch. MIT candidacy was based on earlier studies (Sparks et al., 1974; Helm-Estabrooks et al., 1989; Sparks, 2008) and defined as: non-fluent aphasia (<50 words/min) after a unilateral left-hemisphere stroke, poor language repetition even for single words [Aachen Aphasia Test (AAT; Graetz et al., 1991), subtest repetition ≤100], poorly articulated speech (AAT spontaneous language subscale articulation ≤3), and moderate to good auditory language comprehension (AAT subtest auditory comprehension ≥33; functional comprehension ≥5). Exclusion criteria were: prior stroke resulting in aphasia, bilateral lesion, intensive MIT prior to start of the study, severe hearing deficit, psychiatric history relevant to language communication.

### Intervention

Melodic intonation therapy was given following the American MIT manual (Helm-Estabrooks et al., 1989; Sparks, 2008). The program consisted of several levels with increasing difficulty. At the first level target utterances were short, formulaic phrases (e.g., ‘How are you?’). As the therapy proceeded, the trained utterances became more complex and less frequent in daily life (e.g., ‘a thunderstorm is coming our way’). For each utterance, a melodically intoned pattern was developed, based on the natural prosody of the utterance [see Sparks et al., 1974; Sparks, 2008, and the American manual (Helm-Estabrooks et al., 1989) for more details on the procedure of creating melodically intoned patterns]. Utterances were trained in a hierarchy of steps: patients and SLTs first produced the melodically intoned utterance together, while hand tapping the rhythm of the utterance. Gradually, the SLT provided less support and the intoned pattern was replaced by the normal prosody. The final step consisted of independent spoken production of the target utterance.

The therapy was given by experienced SLTs in nine rehabilitation centers and aphasia centers. All were trained to deliver MIT according to the study protocol (Van der Meulen et al., 2014). The protocol comprised a list of Dutch target utterances for each level, along with their intoned pattern. In addition to this standard set, the SLT and the patient or a close relative developed a set of personally relevant utterances, such as utterances related to hobbies or favorite food. At least 50% of the therapy time had to be spent on training the standard set.

To ensure a treatment intensity of 5 h/week, we developed an iPod application enabling patients to practice independently at home. The minimum amount of face-to-face therapy time was 3 h/week. When the SLT was unable to offer 5 hours of treatment per week, patients used the iPod to practice at home, at least 2 h/week, but no more than 7 h/week. Patients or a close relative recorded the time spent on homework assignments and SLTs recorded therapy time for each session.

In the control condition, no individual treatment was offered. Many of the patients were recruited from aphasia centers, where they participated in aphasia groups, offering opportunities for social interaction, as well as low intensity group therapy to support verbal and non-verbal communication (e.g., written communication, discussing news items). Participation in these groups was allowed in both conditions.

### Outcome Measures

Assessment was done at baseline (T1), 6 weeks later (T2), and 12 weeks later (T3) (**Figure 1**). Outcome measures were: the MIT repetition task, the subtests naming, repetition and auditory comprehension from the AAT (Graetz et al., 1991), the Amsterdam-Nijmegen Everyday Language Test (ANELT; Blomert et al., 1995), and the Sabadel story retell task (Van Eeckhout, 1982). The MIT repetition task was designed for our two MIT trials and comprised 11 trained and 11 untrained matched sentences.

When evaluating the effect of aphasia treatment, it is crucial to distinguish between improvement on trained items (direct effect), improvement on non-trained items (indirect effect), and generalization to functional language use (Van der Meulen et al., 2012; Zumbansen et al., 2014). In this study, we selected outcome measures to evaluate the effect of MIT at different levels: (1) improvement in repeating trained items (MIT repetition task, trained items), (2) generalization to untrained items (MIT repetition task, untrained items and AAT subtest repetition), (3) generalization to word retrieval (AAT subtest naming), (4) further generalization to verbal communication (ANELT and Sabadel). The goal of MIT is to improve verbal fluency and connected speech, i.e., the latter level of success. In addition to these measures for language production, we also included a language comprehension task (AAT subtest auditory comprehension) because several studies have reported improved auditory comprehension after MIT (Sparks et al., 1974; Helm-Estabrooks, 1983; Bonakdarpour et al., 2003).

### Statistical Analysis

Since no data from chronic aphasic patients were available, we performed an *a priori* power analysis based on Sabadel (Van Eeckhout, 1982) data from *subacute* severe non-fluent patients participating in a small pilot study (Paul and Pijnenburg, 2002, unpublished). For this small subacute sample, the effect size was 0.90, considered to be large in terms of Cohen’s *d*. With an α = 0.05, β = 0.20 and power = 0.80, the estimated sample size for this effect size was 15 patients per group.

Because the data were not normally distributed, we used non-parametric tests. Potential differences at baseline between the experimental and the control group were analyzed using Mann–Whitney *U* tests for continuous data and Fisher’s Exact Test for categorical variables.

The efficacy of MIT was evaluated for each outcome measure at T2 by means of univariable regression analyses, adjusted for baseline (T1), with group assignment (experimental vs. control) as the independent variable. Because there was heterogeneity in both groups with regard to the severity of the aphasia, all regression analyses were also adjusted for aphasia severity, as expressed by the score on the AAT Token Test.

We further examined whether, as a group, patients showed language improvement after MIT. For this, we used the Wilcoxon signed rank test to examine change from pre-MIT scores (T1 experimental group, T2 control group) to post-MIT scores (T2 experimental group, T3 control group) in all patients.

We considered the following potential determinants for MIT success: age, gender, aphasia severity (Token Test AAT), treatment intensity, patients’ linguistic profile at the start of MIT: pre-MIT scores on AAT language repetition, naming, auditory comprehension, and the non-verbal Semantic Association Task (Visch-Brink et al., 2005). The influence of these variables on all outcome measures was examined through univariable regression analyses. For these analyses, we used the post-MIT scores of all patients (T2 experimental group, T3 control group) as the dependent variable, adjusted for the pre-MIT scores of all patients (T1 experimental group, T2 control group). Subsequently, scores on all outcome measures were dichotomized into responders (improvement >10 on MIT repetition, >14 on AAT repetition, >16 on AAT naming, >7 on the ANELT, >0 on the Sabadel) and non-responders (Van der Meulen et al., 2014). Mann–Whitney *U* tests and Fisher’s Exact Tests were used to examine group differences.

All analyses were performed on an intention-to-treat basis using SPSS version 21. A level of significance of *p* < 0.05 was used in all analyses.

## Results

### Participants

**Figure 1** presents the flow chart of patient inclusion. Of the 44 chronic aphasic patients referred to the study, 17 (38.6%) were included. The main reason for exclusion was failure to meet the inclusion criteria (*n* = 20). Other reasons were: unwillingness to participate because of the treatment intensity (*n* = 3), travel distance to a center participating in the trial (*n* = 3), or other (*n* = 1). Ten participants were allocated to the experimental group that received MIT first and 7 to the control group receiving MIT after a waiting period of 6 weeks. We were unable to achieve the aim of 15 patients per group. Inclusion took considerably longer than anticipated and, after extending the inclusion period once, no further funding was available. There were no drop-outs in the experimental group. In the control group there was one drop-out after the assessment at T2. This patient did not start MIT for personal reasons.

**Table 1** presents the baseline characteristics of both groups. There were no significant differences between groups.

**Table 1:** Baseline characteristics (*n* = 17).

	Experimental	Control	*p*
	(*n* = 10)	(*n* = 7)	
	Mean (*SD*)	Mean (*SD*)	
Age, years	58.1 (15.2)	63.6 (12.7)	0.67
Gender, % male	70%	57.1%	0.64
Education^a^	4.8 (2.9)	4.3 (2.6)	0.50
Time post stroke, months	33.1 (19.4)	42.6 (23.7)	0.54
Stroke type			
Ischemic, %	80%	100%	
Unknown, %	20%	0%	
Stroke localization, % LH	100%^b^	100%	1.0
Handedness, % right handed^c^	90%	100%	1.0
AAT Token Test	35.4 (10.9)	31.0 (18.7)	1.0
AAT language repetition	53.6 (28.5)	35.3 (22.7)	0.23
AAT auditory comprehension	40.7 (6.4)	41.7 (7.6)	0.89
AAT naming	25.7 (27.8)	16.0 (30.8)	0.30
ANELT	15.7 (6.2)	12.9 (7.1)	0.32
Sabadel, CIUs	4.9 (8.9)	3.3 (8.3)	0.48
MIT repetition	30.7 (26.0)	23.0 (16.8)	0.60
SAT non-verbal	22.9 (5.3)	23.9 (4.8)	0.74


*SD, standard deviation; LH, left hemisphere; AAT, Aachen Aphasia Test; ANELT, Amsterdam-Nijmegen Everyday Language Test; CIU, Correct Information Units; MIT, Melodic Intonation Therapy; SAT, Semantic Association Test.*

*^a^Level of education 1 = lowest (primary school), 8 = highest (university); ^b^one patient was referred to us as aphasic after a large infarct in the left hemisphere. This patient also participated in an fMRI study we were running. The MRI scan showed a large infarct in left fronto-parietal regions as well as a right temporo-parietal infarct. ^c^Handedness before stroke (Edinburgh Handedness Inventory and/or medical information).*

### Efficacy

**Table 2** shows the improvements during the intervention period (T1–T2) for each intervention group, as well as the results of the between-groups analyses.

**Table 2:** Mean scores on all outcome measures (T1 and T2) and group comparisons per outcome measure at T2.

	Experimental group (MIT) (*n* = 10)			Control group (no treatment) (*n* = 7)			Group comparison (MIT versus control)	
			
	T1 Mean (*SD*)	T2 Mean (*SD*)	*p*	T1 Mean (*SD*)	T2 Mean (*SD*)	*p*	β	*p*
Sabadel	4.9 (8.9)	5.8 (7.6)	0.50	3.3 (8.3)	5.8 (14.2)	0.18	-1.77	0.50
ANELT	15.7 (6.2)	16.1 (8.3)	0.91	12.9 (7.1)	13.9 (7.2)	0.32	-0.88	0.58
Naming (AAT)	25.7 (27.8)	28.9 (28.3)	0.21	15.4 (34.3)	18.2 (40.7)	0.32	-0.53	0.90
Repetition (AAT)	53.6 (28.5)	59.7 (33.6)	0.12	35.3 (22.7)	43.3 (23.7)	**0.046**	-3.09	0.61
MIT task: trained items	17.2 (14.5)	30.0 (18.1)	<**0.01**	14.9 (10.4)	14.4 (12.1)	0.75	13.32	**0.02**
MIT task: untrained items	13.5 (11.7)	18.5 (14.2)	**0.03**	8.1 (7.2)	10.3 (9.8)	0.17	1.99	0.40
Auditory comprehension (AAT)	40.7 (6.4)	38.5 (10.3)	0.48	40.7 (7.7)	42.3 (9.5)	0.60	-0.05	0.70


*SD, standard deviation; ANELT, Amsterdam-Nijmegen Everyday Language Test; AAT, Aachen Aphasia Test. Bold values, significant.*

After MIT, the experimental group showed significant improvement on the repetition of both trained and untrained utterances (**Table 2**). No significant improvement was observed on any of the other outcome measures. Unexpectedly, the control group improved on the AAT repetition task (**Table 2**). This observation is difficult to explain, since this group did not receive any language production treatment.

The regression analysis revealed a positive treatment effect on trained items: the experimental group improved significantly more on trained items than the control group (**Table 2**). On all other outcome measures no significant effect of MIT was observed. After adjustment for aphasia severity, as expressed by the score on the AAT Token Test at baseline, outcomes did not change.

### T2–T3

**FIGURE 2:**
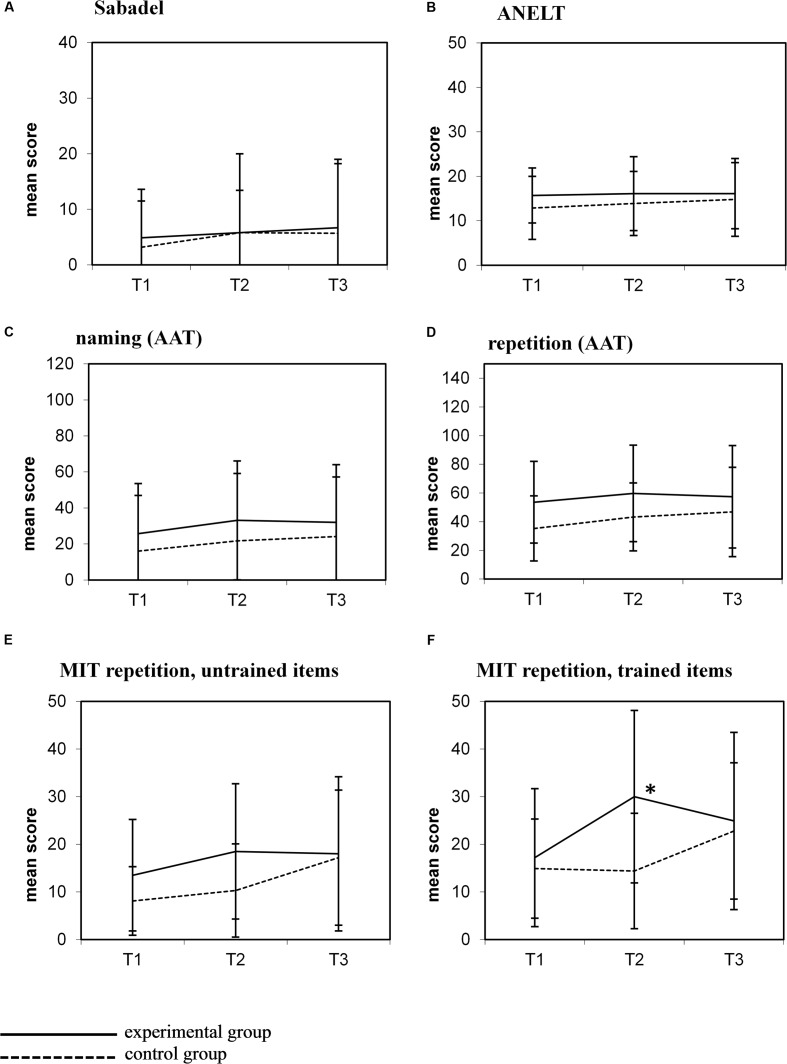
**Mean scores (±1 SD) for all outcome measures at the three test moments.** **(A)** Sabadel; **(B)** ANELT; **(C)** Naming (AAT); **(D)** Repetition (AAT); **(E)** MIT repetition, untrained items; **(F)** MIT repetition, trained items.

Between T2 and T3 the control group received MIT, whereas no individual treatment was given to the experimental group (**Figure 1**). The graphs in **Figure 2** show the mean scores of both groups for all outcome measures at all test moments.

Visual inspection of these graphs shows a similar pattern after MIT in the control group (i.e., between T2 and T3) as observed in the experimental group after MIT (T1–T2): improvement on the MIT repetition task, both on trained and untrained items **Figures 2E,F**), but no improvement on other outcome measures (**Figures 2A–D**). In contrast to the experimental group, this improvement in the control group did not reach significance (trained items: *t* = 1.48; *p* = 0.20; untrained items: *t* = 2.19; *p* = 0.08). There was no difference in MIT intensity in both groups (experimental group mean MIT (T1–T2) 5.01 h/week (*SD* 2.25); control group MIT (T2–T3) 6.04 h/week (*SD* 0.09); *t* = 1.17, *p* = 0.27).

In the experimental group, the beneficial effect of MIT on trained items observed at T2 was not maintained at the follow-up measure 6 weeks later (T3) (**Figure 2F**). Patients performed significantly worse on this task at T3 compared to T2 (*t* = -2.29; *p* = 0.049).

**Table 3** shows the language improvement after MIT of all patients (T1, T2 for the experimental group; T2, T3 for the control group). As a group, patients improve significantly on both trained and untrained items after MIT.

**Table 3:** Improvement after MIT for all patients (*n* = 16).

	Pre-MIT Mean (*SD*)	Post-MIT Mean (*SD*)	*p*
Sabadel	5.6 (11.2)	5.8 (9.3)	0.87
ANELT	15.2 (6.6)	15.6 (8.0)	0.67
Naming (AAT)	25.8 (31.7)	27.3 (30.5)	0.37
Repetition (AAT)	49.6 (27.2)	54.9 (32.3)	0.09
MIT task: trained items	16.8 (13.3)	27.3 (16.7)	<**0.01**
MIT task: untrained items	12.8 (10.8)	18.0 (13.7)	<**0.01**
Auditory comprehension (AAT)	41.1 (7.7)	38.8 (9.4)	0.17


*SD, standard deviation; ANELT, Amsterdam-Nijmegen Everyday Language Test; AAT, Aachen Aphasia Test. Bold values, significant.*

### Responders and Non-responders

**Table 4** presents individual data on all outcome measures.

**Table 4:** Improvement after MIT (Δ post-MIT–pre-MIT) on each outcome measure per patient.

Patient nr \ Task	Sabadel, CIUs	ANELT	Naming (AAT)	Repetition (AAT)	MIT task: untrained items	MIT task: trained items
1	1^∗^	2	-	-8	10	24^∗^
2	9,5^∗^	2	13	21^∗^	5	36^∗^
3	-2	2	9	10	6	16^∗^
4	0	0	-2	10	3	6
5	3^∗^	-2	4	17^∗^	1	4
6	0	0	4	-5	6	-9
7	-7	1	-6	18^∗^	15^∗^	11^∗^
8	0	0	0	6	0	0
9	-6,5	8^∗^	-	26^∗^	11^∗^	16^∗^
10	0	0	2	-4	-2	4
11	0	-2	-	-4	9	24^∗^
12	0	0	0	4	0	0
13	0	0	3	-3	13^∗^	15^∗^
14	0	0	-	-13	-1	12^∗^
15	0	0	0	4	-1	8
16	5^∗^	-4	-9	5	8	1


*^∗^Significant improvement; -, missing; AAT, Aachen Aphasia Test; ANELT, Amsterdam-Nijmegen Everyday Language Test; CIU, Correct Information Units; MIT, Melodic Intonation Therapy.*

As can be seen in this table, there is a large amount of individual variation, with some patients showing no language improvement at all (patients 4, 6, 12, and 14) while others benefit from MIT. Further, after MIT, repetition of trained items improved in 8 out of the 16 participating patients, but generalization to untrained items or functional language only occurred in a small subset of these patients (for instance patients 2, 7, and 9). No significant differences between responders and non-responders were observed.

### Determinants

Treatment intensity (face-to-face treatment with a speech language therapist combined with home work assignments) was the only variable that was significantly related to improvement on trained items (β = 0.06, *p* = 0.03). Higher intensity yielded greater improvement on trained items. None of the other variables was significantly related to MIT success.

## Discussion

This is the first RCT examining the efficacy of MIT in the chronic phase after stroke. We investigated its efficacy at several levels, and found that, as a group, patients improved on both trained and untrained items after MIT. When language improvement after MIT in the experimental group was compared to language improvement in the untreated control group, MIT appeared to be only effective on the repetition of trained material, without generalization effects to untrained material, word retrieval or verbal communication in daily life. This effect was transient: 6 weeks after finishing MIT, patients had been unable to maintain their MIT-related language gains.

The study was, however, underpowered and the results therefore have to be considered as preliminary. The study might be seen as a pilot RCT and confirmation from larger RCTs is required to verify our results. This is all the more important, because our results contrast with findings from previous studies, in which long term improvement of naming and verbal communication was reported (Bonakdarpour et al., 2003; Schlaug et al., 2008, 2009; Hough, 2010; Stahl et al., 2013; Wan et al., 2014). Also contrary to some studies (Sparks et al., 1974; Helm-Estabrooks, 1983; Bonakdarpour et al., 2003) we found no effect of MIT on auditory verbal comprehension. There are several possible explanations for these differences. First of all, the lack of generalization effects might be due to the small sample size and the unequal number of patients in the two groups. We used the design of a RCT, but because the study was underpowered, potential positive effects of MIT might have remained unnoticed. Alternatively, the results of our study, albeit small, suggest that MIT has only a limited effect in chronic aphasia. The previous case studies show that individual chronic aphasic patients do benefit from MIT, but at the group level, its effect is small and temporary. Note that there is a considerable inter-subject variation in our study, with some patients obtaining substantial gains on functional tasks, while others did not benefit at all from MIT.

This large variation in improvement after MIT raises the question for which patient MIT is best suited. All participants in our study fitted the criteria for MIT-candidacy as defined in the literature (Sparks et al., 1974; Naeser and Helm-Estabrooks, 1985; Helm-Estabrooks et al., 1989; Sparks, 2008). Nevertheless, within this well-defined group, patients’ responses to MIT differed. In order to implement MIT more effectively, stricter criteria are needed. We were, however, unable to find any patient-related variables that were significantly related to MIT success. Hence, the question which patients benefit most from MIT remains open.

The only variable significantly related to improvement after MIT was treatment intensity. More intensive training yielded larger improvement on trained items. This is in line with other studies and reviews showing that higher intensity of aphasia therapy yields larger language improvement (Robey, 1998; Bhogal et al., 2003; Bakheit et al., 2007b; Brady et al., 2012). It is possible that a longer and more intensive treatment would have yielded generalization to verbal communication. However, not all aphasic patients are able to engage in an intensive language production therapy for such a long period of time. The latest Cochrane review on aphasia therapy showed that studies with high intensity treatment have a larger number of drop-outs than studies in which therapy is provided less frequently (Brady et al., 2012). In our study, there were no drop-outs, suggesting that following MIT in this intensity is feasible, even in severe non-fluent aphasic patients.

The optimal timing of aphasia therapy is an important topic in current aphasia rehabilitation research. Although several studies point to a larger effect of aphasia therapy if applied in the first 3 months after stroke, (Robey, 1998; Bakheit et al., 2007a) a recent review showed that the currently available evidence on the optimal timing of aphasia therapy is inconclusive and insufficient (Nouwens et al., 2015). The contradictory findings may be related to differences in study populations, e.g., including persons with various types of aphasia and/or receiving different types of aphasia treatment. Studies examining the efficacy of one specific treatment at different stages post stroke are rare. We investigated the effect of MIT in the subacute (Van der Meulen et al., 2014) and chronic stages post stroke, using similar study designs and similar outcome measures, allowing a comparison of the results of both studies. The beneficial effects of MIT observed in the present study with chronic stroke patients are less favorable than the effects in the subacute stage post stroke, where MIT yielded improved repetition of trained as well as untrained items, with a trend to improved verbal communication (Van der Meulen et al., 2014). This difference has to be interpreted cautiously. It may suggest that MIT is more effective in the subacute stage post stroke. However, our data do not allow statistical analyses comparing the effect of MIT in both stages post stroke. Carefully designed studies with large sample sizes are needed to determine the optimal timing of MIT.

The small sample size of this study is a clear limitation and confirmation from larger studies is required. Further, we were unable to collect data on the size and location of the lesion of each participant as well as on the severity of their stroke. It has been suggested that these variables are related to MIT success (Lazar et al., 2008; Schlaug et al., 2008, 2009; Merrett et al., 2014). Hence, this information might have led to a better definition of MIT candidacy.

## Conclusion

The results of this study suggest that the effect of MIT in chronic severe non-fluent aphasia is limited. Although the effect is small and generalization to functional language use could not be demonstrated, its impact should not be underestimated: being able to say the name of one’s partner or to ask for a drink can represent a considerable improvement in the quality of life of someone who, before MIT, was unable to utter any intelligible words. At the same time, these results indicate that the expectations related to MIT in chronic aphasia should not be raised too high.

## Author Contributions

IV has contributed substantially to the design of the study, conducted the research (data acquisition, analysis and interpretation of the data). MV, MH, EV-B, and GR have contributed substantially to the design of the study, the data analysis and interpretation. They have revised earlier versions of the manuscript critically. All authors approve this version to be published and agree to be accountable for all aspects of the work.

## Conflict of Interest Statement

IV and MV authored a Dutch version of the MIT treatment program after completion of the MIT effect studies (Van der Meulen et al., 2015). The publisher had no influence on the data collection, methods, interpretation of the data, and final conclusions. Rijndam rehabilitation Institute will receive revenues of the manual. All the other authors declare that the research was conducted in the absence of any commercial or financial relationships that could be construed as a potential conflict of interest.
